# Supplementary material for: Continuous Electrical Stimulation Affects Initial Growth and Proliferation of Adipose-Derived Stem Cells
Source: Biomedicines. 2020 Nov 8;8(11):482. doi: 10.3390/biomedicines8110482 (PMC7695310; doi:10.3390/biomedicines8110482)
Supplement: Supplementary file 1 [file biomedicines-08-00482-s001.pdf]

Supplementary data

1

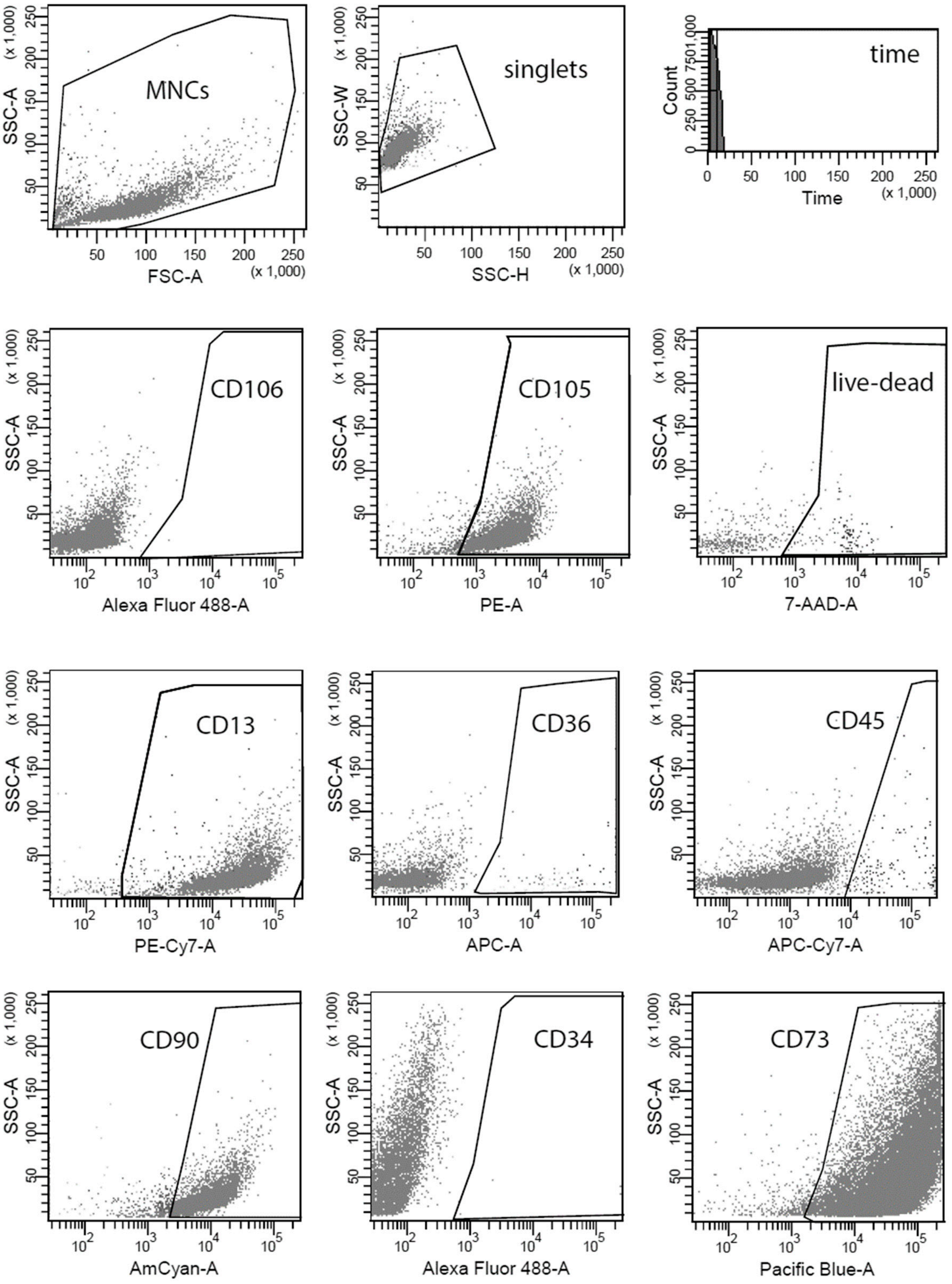

2

**Surface protein**	**[%] of viable singlets**<br>Mean value, n=3	Standard Deviation, SD
**CD13**	98.10	0.75
**CD45**	1.95	0.92
**CD90**	85.50	11.38
**CD34**	0.11	0.16
**CD73**	96.17	4.54
**CD105 (Endoglin)**	84.87	8.01
**CD36**	1.54	1.09
**CD106 (VCAM-1)**	0.34	0.30

Supplementary Figure 1. Surface marker expression in human adipose -derived mesenchymal stem cells (ADSC) detected by flow cytometry. **(A)** Representative illustration of a flow cytometric analysis of ADSC at passage 8 using mesenchymal and hematopoietic stem cell markers. Singlets (dot plot SSC-W versus SSC-H) from mononuclear cells (MNCs, plot SSC-A versus FSC-A) and negative-expressing cells for 7-AAD were included for further analysis. Mesenchymal stem cell markers (CD105, CD13, CD90, CD73) were present whereas hematopoietic stem cell markers (CD34, CD45) as well as bone marrow specific antigen CD36 were hardly to detect. **(B) Means of p**ercentages of the corresponding CD's of viable singlets. n=3 (ADSC were taken from passages 8(n=2) and 9 (n=1))
